# Hemoglobin Lansing (Alpha) [HBA2 CD87 (HIS>GLU) (C>A)] in a Turkish Individual Resulting from Another Nucleotide Substitution

**DOI:** 10.4274/tjh.2014.0102

**Published:** 2014-09-05

**Authors:** Nejat Akar, Didem Torun, Ayşenur Öztürk

**Affiliations:** 1 TOBB-ETU Hospital, Clinic of Pediatrics, Ankara, Turkey; 2 Ankara University Faculty of Medicine, Department of Pediatric Molecular Genetics, Ankara, Turkey

**Keywords:** Hb Lansing, abnormal hemoglobin, Alpha-globin

## TO THE EDITOR

Several hemoglobin variants, including novel ones, have been reported in the Turkish population [1,2,3]. Herein, we describe a novel nucleotide alteration of the alpha-2 chain variant, hemoglobin (Hb) Lansing CD87 (HIS>GLU).

The index case was a 21-year-old Turkish woman living in Ankara. She was admitted to the TOBB-ETU University Pediatrics Outpatient Department for pre-marital counseling. Her physical examination was normal. Hemoglobin, hematocrit, and MCV values were 13.1 g/dL, 42.7%, and 95 fL, respectively. Levels of Hb A1, Hb A2, and Hb X were observed as 65.5%, 1.88%, and 22.24%, respectively, with high-pressure liquid chromatography. Written informed consent for genetic analysis was obtained from the patient. 

DNA was isolated from a peripheral blood sample with the phenol-chloroform protocol. All of the exons of the HBB, HBA1, and HBA2 genes were amplified by polymerase chain reaction (PCR). The entire coding and intronic sequences of the alpha-1 and alpha-2 globin genes were amplified as one amplicon each. While the forward primer was the same for the 2 genes, the reverse primers were specific to the alpha-1 and alpha-2 genes. These amplicons were sequenced using internal primers as described previously [4,5]. PCR products were cleaned with a PCR purification kit (Roche, Germany) and then samples were sequenced using an automatic DNA Sequencer (Beckman Coulter, USA). The DNA was also tested for the -α3.7, -α4.2, -MED, and -α20.5 deletions using multiplex PCR according to the described methods [6,7].

**Figure 1:**
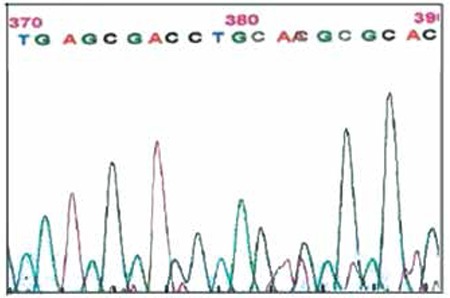
HbA2 gene 264 C>A transition at exon 2. 84 85 86 87 88 89

A novel missense mutation was found in codon 87 (CAC>CAA) in the heterozygous state. The sequencing data showed the variant as a mutation at codon 87 of C changing to A, leading to histidine substitution to glutamine, which was not previously described (Figure 1).

There is only one mutation reported at codon 87 of the alpha-2 gene by Sarikonda et al. They reported a missense mutation at alpha-2 gene codon 87 with substitution of CAC to CAG (His>Gln) and they named this variant Hb Lansing [5]. The reported patient showed the heterozygous form and was asymptomatic with low pulse oximeter readings in the index case. Arterial blood gas showed an O2 saturation of 98% on room air [8]. The proportion of the variant in family members was between 10.6% and 24.2% [6]. Our patient was also asymptomatic. However, we did not have the chance to further investigate the patient for these above-mentioned clinical symptoms. Other than this, in Asia, Ishitsuka et al. reported a case of Hb Lansing detected by false low oxygen saturation on pulse oximetry [7].

The variant that we describe is identical in its protein structure to Hb Lansing. In both cases, the nucleotide change in codon 87 leads to a glutamine residue: CAA in our case and CAG in Hb Lansing. In human coding regions, CAG is much more common in terms of frequency of usage per thousand (32.95) and relative frequency among synonymous codons (0.73). For CAA, these values are 11.94 and 0.27, respectively. Therefore, this case is described with codon usage bias [8]. A similar situation was also reported previously in the case of Hb Niigata. Hb Niigata is an alteration of valine to leucine at beta-globin gene codon 1 with a nucleotide change of G>C/T, which was reported in a Japanese and a Romanian. Moradkhani et al. named the variant of the Romanian individual Hb Niigata (C) [9,10]. Located in different genes, the Hb Niigata and Hb Lansing variants can be explained by the wobble hypothesis. According to the wobble hypothesis, base pairs are relaxed at the third position, so a base can pair with more than one complementary base. In these variants, changes occur in the third base (CAA>CAG) [11].

As Hb Lansing was reported previously, we named this new variant Hb Lansing (A).

Since Turkey is located at the intersection of 3 continents, it is not surprising that many different hemoglobin variants are observed.

## CONFLICT OF INTEREST STATEMENT

The authors of this paper have no conflicts of interest, including specific financial interests, relationships, and/ or affiliations relevant to the subject matter or materials included.
